# Multicomponent gold nano-glycoconjugate as a highly immunogenic and protective platform against *Burkholderia mallei*

**DOI:** 10.1038/s41541-020-00229-9

**Published:** 2020-09-10

**Authors:** Daniel Tapia, Javier I. Sanchez-Villamil, Alfredo G. Torres

**Affiliations:** 1 Department of Microbiology and Immunology, University of Texas Medical Branch, Galveston, TX 77550 USA; 2 Department of Pathology, University of Texas Medical Branch, Galveston, TX 77550 USA

**Keywords:** Microbiology, Bacteria, Bacteriology, Vaccines, Conjugate vaccines

## Abstract

*Burkholderia mallei* (*Bm*) is a facultative intracellular pathogen and the etiological agent of glanders, a highly infectious zoonotic disease occurring in equines and humans. The intrinsic resistance to antibiotics, lack of specific therapy, high mortality, and history as a biothreat agent, prompt the need of a safe and effective vaccine. However, the limited knowledge of protective *Bm*-specific antigens has hampered the development of a vaccine. Further, the use of antigen-delivery systems that enhance antigen immunogenicity and elicit robust antigen-specific immune responses has been limited and could improve vaccines against *Bm*. Nanovaccines, in particular gold nanoparticles (AuNPs), have been investigated as a strategy to broaden the repertoire of vaccine-mediated immunity and as a tool to produce multivalent vaccines. To synthesize a nano-glycoconjugate vaccine, six predicted highly immunogenic antigens identified by a genome-wide bio- and immuno-informatic analysis were purified and coupled to AuNPs along with lipopolysaccharide (LPS) from *B. thailandensis*. Mice immunized intranasally with individual AuNP-protein-LPS conjugates, showed variable degrees of protection against intranasal *Bm* infection, while an optimized combination formulation (containing protein antigens OmpW, OpcP, and Hemagglutinin, along with LPS) showed complete protection against lethality in a mouse model of inhalational glanders. Animals immunized with different nano-glycoconjugates showed robust antigen-specific antibody responses. Moreover, serum from animals immunized with the optimized nano-glycoconjugate formulation showed sustained antibody responses with increased serum-mediated inhibition of adherence and opsonophagocytic activity in vitro. This study provides the basis for the rational design and construction of a multicomponent vaccine platform against *Bm*.

## Introduction

Glanders is a zoonotic disease with a high degree of transmissibility to humans and mortality in solipeds (e.g., horses, mules, and donkeys). The infection is caused by the Gram-negative pathogen *Burkholderia mallei* (*Bm*)^1–3^. This historic biothreat pathogen has been intentionally used as a biological weapon a number of times because of its high transmissibility, mortality, and amenability for aerosolization^3–5^. In addition, *Bm* is a non-motile intracellular pathogen with a complex intracellular lifestyle that uses a myriad of secreted effectors to allow survival and evasion of host immune responses^3,4^. Due to the pathogen’s inability to survive for long periods of time outside its mammalian host, *Bm* is thought to have evolved by reductive evolution from its genetically related counterpart, *Burkholderia pseudomallei* (*Bpm*), the causative agent of melioidosis^6,7^. Both of these pathogens are capable of infecting and surviving inside phagocytic and non-phagocytic cells, including lung epithelial cells^1,7^. Depending on the route of infection, by either percutaneous inoculation (also known as farcy) or inhalation (glanders), humans and equines can present a wide range of clinical signs and symptoms^6^. Pulmonary infection with *Bm* can be developed as either acute or chronic infections^6,8^. Its aerosolization properties together with the ability of infecting both humans and animals, has resulted in the dual classification of *Bm* as a Tier 1 Select Agent by the Centers for Disease Control and Prevention (CDC) and the United States Department of Agriculture (USDA)^4^.

*Bm* and *Bpm* are naturally resistant to a wide range of antibiotics, and the available ones are limited or require extensive treatment regimens^1,5^. Furthermore, *Bm*-specific therapeutic interventions have not gained much interest in the last ten years and fewer vaccine candidates have been reported for *Bm*, in comparison to *Bpm*^2,9^. Therefore, there is a need to identify and develop specific preventive measures for each of these pathogens for which no vaccine is currently available. Although glanders was eradicated from the Western Hemisphere in the late 20^th^ century; today, glanders remains largely an occupational hazard for individuals that come in close contact with infected animals, including endemic areas in Western Asia, India, Africa, and South America^6,8^. Although a variety of vaccine candidates have been tested, live attenuated vaccines have provided the best evidence of complete protection and near-sterilizing immunity^10,11^. One outstanding concern associated with the live-attenuated vaccine approaches is their safety, especially if an effective vaccine will be used in immunocompromised individuals. Further, many other vaccines have failed to define the biomarkers of protection or the vaccine-mediated immune protective mechanisms. Therefore, alternative vaccination strategies, such as subunit vaccines, have to overcome the obstacles of conventional vaccine platforms to surmount the challenges of inducing robust humoral immune responses and engaging cellular immune responses^12^.

Nanoparticle formulations offer attractive features that dictate and direct the immune response, including protection of the antigen from degradation, facilitating antigen movement across the epithelia, uptake and processing by antigen-presenting cells (APCs), depot formation, and co-delivery of antigens^12,13^. Given that the processes that govern antigen uptake and presentation by APCs are dependent on particle characteristics, nanovaccines offer a means of augmenting the immunogenicity of subunit candidates that could result in increased protection. Gold nanoparticles (AuNPs) represent a platform with documented evidence of safety in a number of models, including infectious disease and cancer, as well as their use as effective nano-delivery systems which utilizes a number of mechanisms, including increase antigen uptake and processing, resulting in robust protective immune responses^14–17^. The goal of the current study was to exploit the use of the AuNP platform to augment the protective ability of *Burkholderia* antigens that were identified using bio- and immuno-informatic analysis and testing those vaccines in a murine model of glanders. We conjugated each antigen along with lipopolysaccharide (LPS) of *B. thailandensis* (*Bth*), a tested immunogen, to elicit robust humoral responses to all these multiple antigenic molecules and test their protective properties either alone (AuNP-protein-LPS), or as a combination antigenic formulation (AuNP-Combo-LPS) after intranasal delivery. We tested whether single AuNP-protein-LPS formulations afford variable degrees of protection, and if a combination AuNP-glycoconjugate formulation improved the degree of protection against a lethal *Bm* challenge. Further, we also tested if serum from the AuNP-protein-LPS formulations showed antigen-specific responses against protein and LPS and whether this immune response was associated with a profile of anti-*Bm* activity including adherence inhibition to lung epithelial cells and opsonophagocytic activity in macrophages. Our data highlights the identification of three protective antigenic vaccine candidates against *Bm* and reinforced the use of AuNPs as attractive antigen-delivery vehicle to augment protective immune responses.

## Results

### Design and synthesis of gold (AuNP) nanoglycoconjugate vaccine platform

**Fig. 1:**
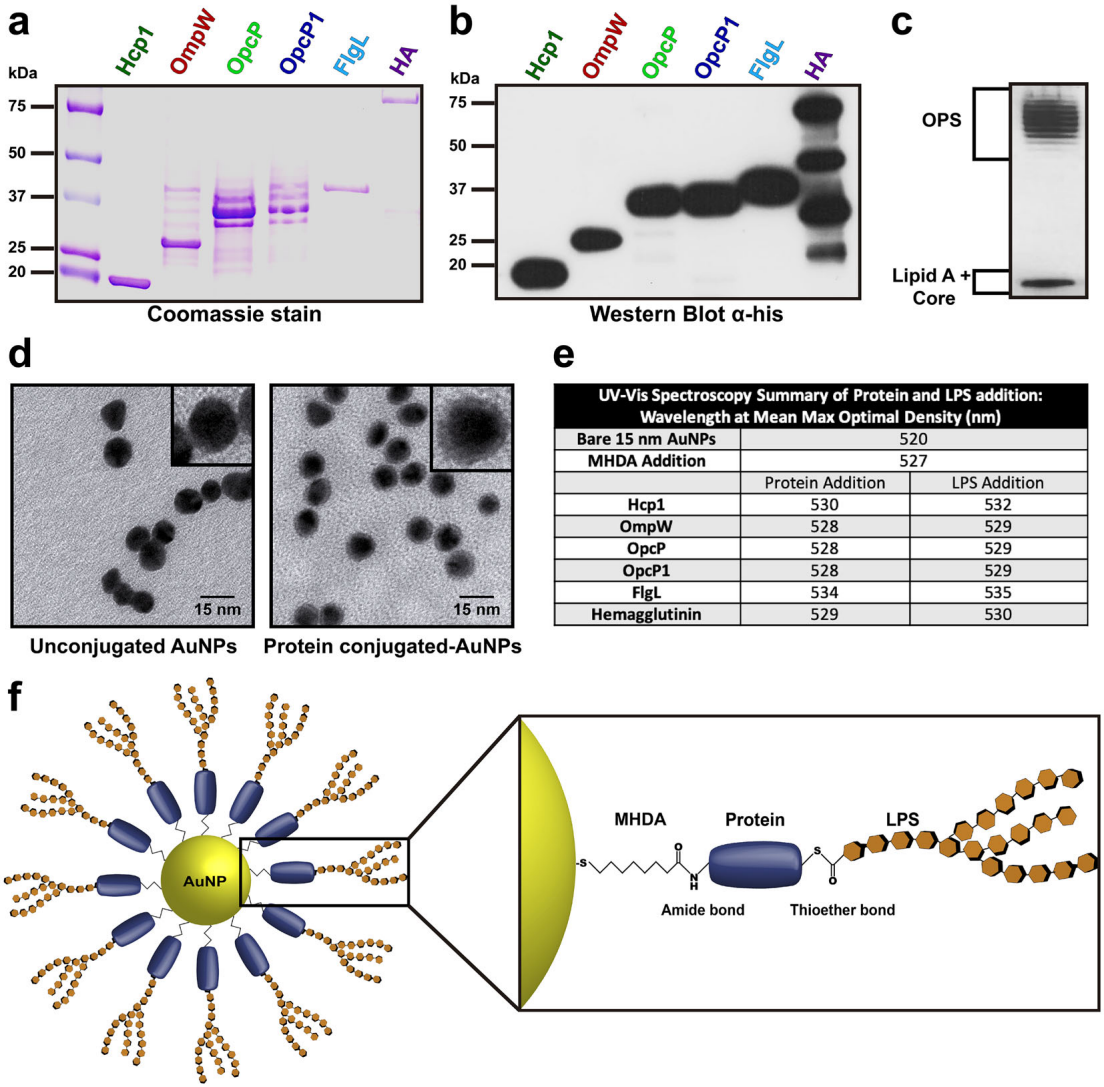
Design and synthesis of gold nanoparticles (AuNP)-coupled as glycoconjugates to bio-and immuno-informatically identified candidates. **a** Coomassie-stained SDS-PAGE gel and **b** Western blot (WB) of recombinantly expressed protein antigens after purification, using 10 μg or 0.25 μg, respectively, of each individual candidate: Hcp1 (18 kDa), OmpW (29 kDa), OpcP (39 kDa), OpcP1 (41.6 kDa), FlgL (42 kDa), and Hemagglutinin (HA) (79.6 kDa). WB and Coomassie-stained gel were generated from the same experiment and processed in parallel. **c** Silver-stained gel of *B. thailandensis* lipopolysaccharide (2 μg) showing the separation of the lipid A and core segments from the O-antigen. **d** Transmission electron micrographs of bare spherical 15 nm AuNPs prior to conjugation and representative particles after conjugation to 5 μg/mL of OmpW. Scale bar 15 nm. Top right inlet shows a magnified view of each particle. **e** UV–Vis spectroscopy showing the Mean Maximum Wavelength (nm) of bare AuNPs, after the addition of 16-mercaptohexadecanoic acid (MHDA) linker, the addition of each protein, and after conjugation to LPS. **f** Graphical scheme of AuNP-linked glycoconjugate showing the amide bond between protein and MHDA linker after carbodiimide synthesis, and the thioether bond between LPS and protein after thiolmaleimide synthesis.

Using a previously published immuno- and bio-informatic prediction model for the identification of predicted antigens conserved between *B. pseudomallei* and *B. mallei*, we selected candidates based on their desirable physicochemical properties and predicted antigenicity and narrowed it to six candidates based on their amenability for purification^18^. These candidates were recombinantly expressed in *E. coli* BL21 and purified by affinity chromatography where all proteins showed undetectable endotoxin levels. All six histidine (His)-tagged proteins were visualized on an SDS-PAGE gel followed by Coomassie staining (Fig. 1a) and by western blot (Fig. 1b, Supplementary Fig. 1). To synthesize the glycoconjugate moiety on the surface of AuNPs, we purified the lipopolysaccharide (LPS) of *B. thailandensis* (*Bth*) and showed a high purity yield by SDS-PAGE gel electrophoresis followed by silver stain (Fig. 1c). Consistently spherical 15 nm-diameter AuNPs were synthesized using the Turkevich method^19^, and visualized by transmission electron microscopy, before and after conjugation to proteins (Fig. 1d). The protein candidates were immobilized on the AuNP surface by the addition of 16-mercaptohexaundecanoic acid (MHDA), which is a small linker with a thiol group that readily binds AuNPs and possesses a carboxylic acid on the distal end involved in conjugation by carbodiimide synthesis. We covalently coupled *Bth* LPS on protein-decorated AuNPs by thiol-maleimide synthesis (Fig. 1d, e). To confirm the conjugation, we used UV–visible light (UV–vis) spectroscopy to measure the difference in wavelength displacement indicating a red-shift, as seen after the addition of every protein and subsequent addition of LPS (Fig. 1e). This red shift (2 nm difference on average) after the addition of proteins onto the AuNPs allowed us to characterize the stable coupling of protein (amide bond) and LPS (thioether bond) complex onto the surface of the AuNP platform for subsequent in vivo studies (Fig. 1f).

### Intranasal delivery of gold-nanoglycoconjugates protect against lethal inhalational glanders

**Fig. 2:**
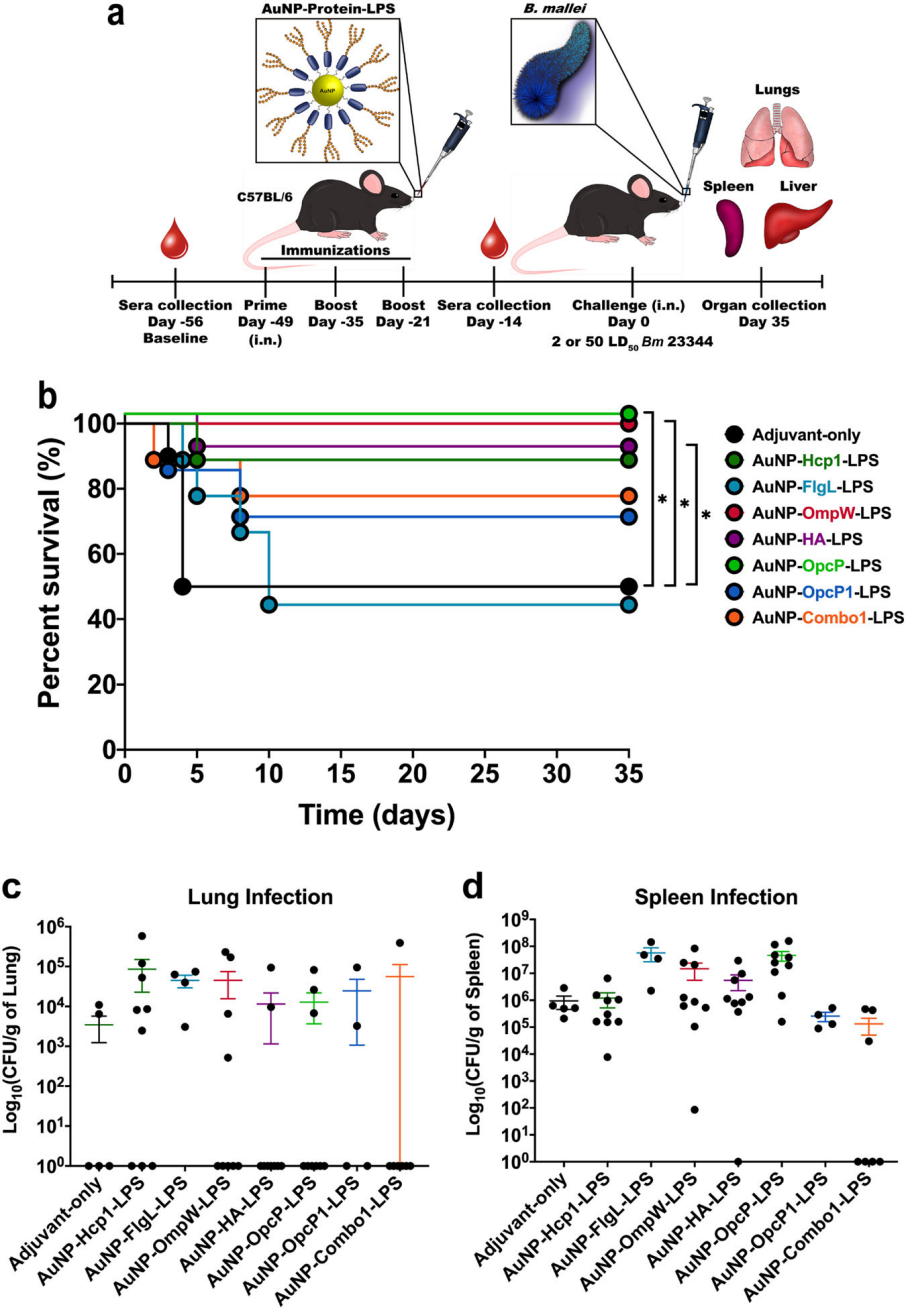
Increased survival from AuNP-OmpW-LPS, -HA-LPS, and OpcP-LPS after low-dose challenge with *Bm* 23344. **a** Graphical illustration of the vaccination timeline. C57BL/6 mice (*n* = 9) were immunized intranasally with formulations containing 10 μg of protein, 10 μg of LPS, and 20 μg of CpG ODN 2395, 3× in two-week intervals. Combination formulation included equivalent amounts of protein (Hcp1, FlgL, OmpW, HA, OpcP, and OpcP1) from each candidate for a total of 10 μg of protein. **b** After intranasal challenge with 2 LD_50_ (2.8 × 10^4^ CFU per mouse) of *Bm* 23344, the **c** lungs and **d** spleens of surviving animals were collected at 35 days post-infection to perform bacterial enumeration. Bacterial load was determined per gram of tissue, and representative panels for colonization are shown in log scale. All colonization data are shown as means ± standard errors of the means (SEM) of results determined per group. Statistical analyses were determined by the Kaplan-Meier method, followed by log-rank test. Levels of significance compared to the adjuvant-only group: *p < 0.05.

To test the protective properties of the different candidates when delivered as vaccines, we used AuNPs alone (AuNP-protein-LPS), or in combination (AuNP-Combo1-LPS), and immunized C57BL/6 mice intranasally using a prime and two-boost vaccination strategy during two-week intervals (Fig. 2a). Each formulation contained a final concentration of 10 μg of protein and 10 μg of LPS coupled to AuNPs and 20 μg of CpG, (TLR9 agonist, as adjuvant) in a final volume of 50 μL. A group of animals also received a combination formulation containing equivalent amounts of each individually conjugated antigen [~1.67 μg/protein (Hcp1, OmpW, OpcP, OpcP1, FlgL, and HA)] along with LPS (AuNP-Combo1-LPS) and CpG. Two weeks after receiving the last immunization, animals were bled to evaluate antibody responses to individual antigens (Fig. 2a). Using a low-dose challenge model, animals were challenged with 2 Lethal Dose-50 (LD_50_) of wild type (WT) *Bm* 23344 three-weeks after receiving the last immunization and to evaluate protection differences between the different antigens (Fig. 2a). Animals immunized with AuNP-OmpW-LPS or AuNP-OpcP-LPS had a significant increase (100% survival) in survival at 35 days post infection (dpi), compared with animals that received an adjuvant-only formulation (Fig. 2b). In addition, animals immunized with AuNP-Hemagglutinin (HA)-LPS or AuNP-Hcp1-LPS displayed a significant increase (90% survival) in survival compared to adjuvant-only treated mice (Fig. 2b). In contrast, only partial protection was provided by the AuNP-OpcP1-LPS (70%), AuNP-Combo1-LPS (80%), or AuNP-FlgL-LPS (40%) formulations (Fig. 2b). After 35 days post infection, surviving animals were euthanized and the lungs and spleens were analyzed for bacterial load. We observed that most animals immunized with single AuNP-protein-LPS formulations had significant colonization in the lung with approximately 10^3^ CFU/gram of tissue (Fig. 2c). However, we also found that most animals immunized with the AuNP-Combo1-LPS formulation (6 of 7) had no recoverable bacteria in the lung, except for one mouse (no significant differences) (Fig. 2c). In addition, most animals that survived to 35 days post infection had bacterial colonization in the spleen (no significant difference); however, 60% of animals immunized with the AuNP-Combo1-LPS formulation had no recoverable bacteria (Fig. 2d).

**Fig. 3:**
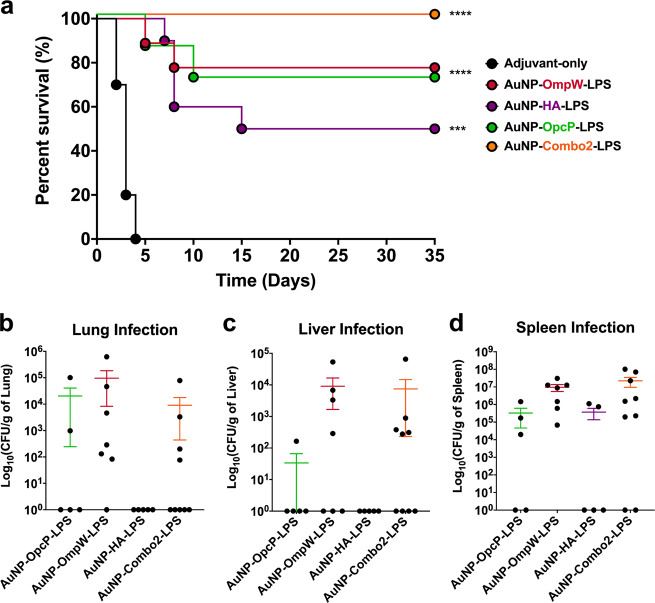
Increased protection by AuNP-Combo-LPS, -OmpW-LPS, -HA-LPS, and OpcP-LPS against high-dose challenge with *Bm* 23344. C57BL/6 mice (*n* = 9) were immunized as described. The AuNP-Combo-LPS vaccinated group contained equivalent amounts of protein (OmpW, HA, and OpcP) from each candidate for a total of 10 μg of protein. **a** After intranasal challenge with 50 LD_50_ (7 × 10^5^ CFU per mouse) of *Bm* 23344, the **b** lungs, **c** livers, and **d** spleens of surviving animals were collected at 35 days post-infection and bacterial enumeration performed. Bacterial load was determined per gram of tissue, and representative panels for colonization are shown in log scale. All colonization data are shown as means ± standard errors of the means (SEM) of results determined per group. Statistical analyses were determined by the Kaplan-Meier method, followed by log-rank test. Levels of significance compared to the adjuvant-only group: ***p < 0.0005, ****p < 0.0001.

To further increase the protection afforded by the candidates with the highest percent survival in the low dose-challenge, we conducted an in vivo protection study focusing on antigens OmpW, OpcP, HA, and a combination of these three (AuNP-Combo2-LPS). Using a similar vaccination strategy as depicted in Fig. 2a, animals were immunized with single protein-LPS formulations, and a formulation containing equivalent amounts of OmpW, OpcP, or HA (~3.33 μg/protein) (AuNP-Combo2-LPS). Three-weeks after receiving the last vaccination, animals were challenged intranasally with 50 LD_50_ of *Bm* 23344 (Fig. 3a). Animals immunized with AuNP-Combo2-LPS displayed 100% survival compared with the adjuvant-only control after 35 dpi, while animals immunized with AuNP-OpcP-LPS, AuNP-OmpW-LPS, or AuNP-HA-LPS, showed 80%, and 50% survival, respectively (Fig. 3a). Surviving animals from each group were evaluated for bacterial infection in the lung, liver, and spleen (Fig. 3b–d). Animals immunized with AuNP-HA-LPS had no recoverable bacteria in either the lung or liver (Fig. 3b, c). Furthermore, 60% of animals immunized with this candidate vaccine did not show any recoverable bacteria in the spleen (Fig. 3d). In addition, 80 and 44% of animals vaccinated with the AuNP-Combo2-LPS did not show any recoverable bacteria in the lung and liver, respectively (Fig. 3b, c). Nonetheless, the highest colonization was found in the spleen of those surviving animals receiving the AuNP-OmpW-LPS formulation (Fig. 3d). These results indicate that the different AuNP-glycoconjugate formulations protect mice against a lethal inhalational challenge with *Bm*, being the highest protection the one provided by the optimized nanovaccine combination (AuNP-Combo2-LPS).

**Fig. 4:**
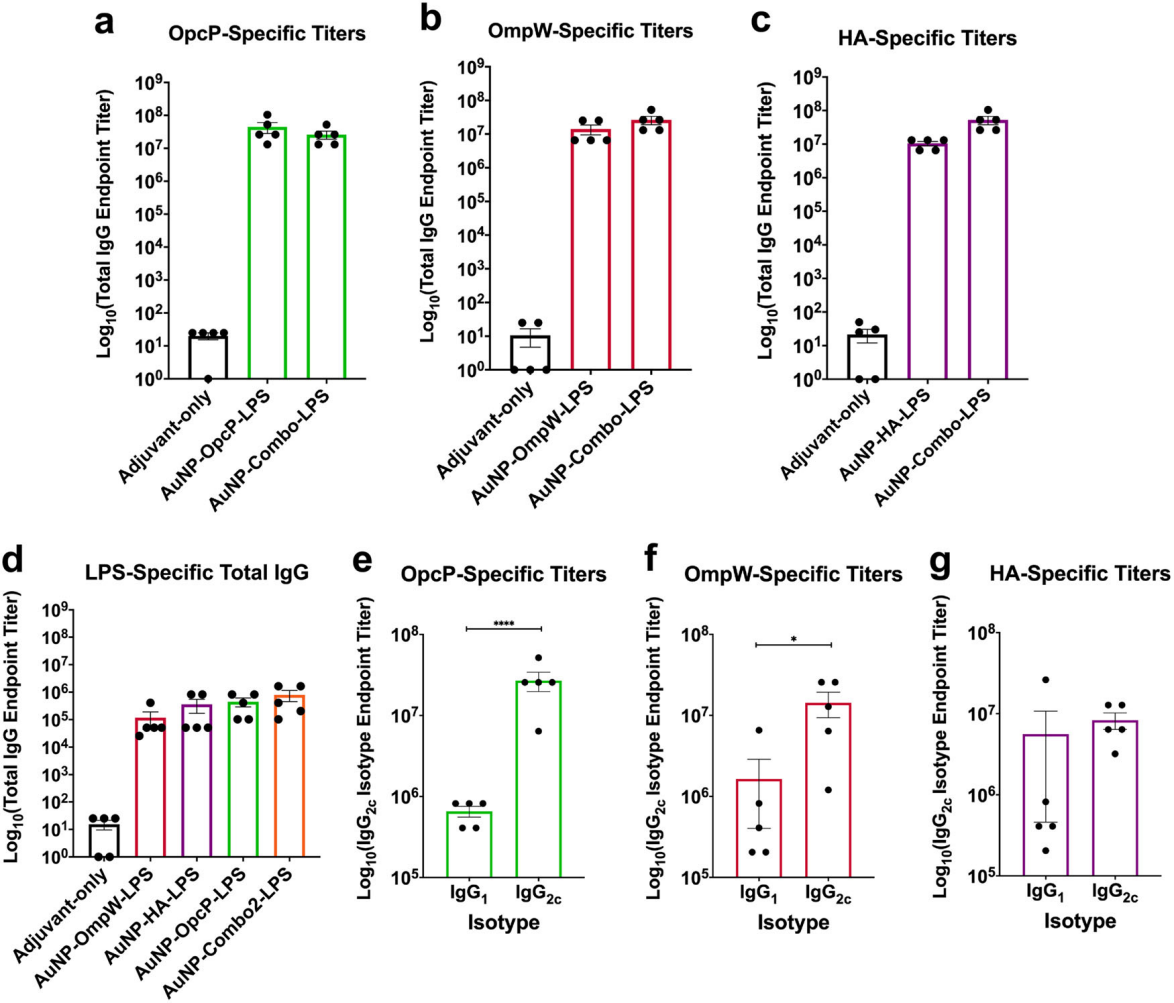
Sustained antibody responses after intranasal delivery of AuNP-nano-glycoconjugate immunization. **a** OpcP-, **b** OmpW-, **c** HA-, and **d** LPS-specific total IgG titers were assessed by ELISA, with endpoint titers defined as twice the standard deviation (SD) of the levels measured for naive sera. Sera samples taken from the mice immunized with the combination formulation (OpcP, OmpW, and HA) were used to assay protein-specific total IgG antibody titers. **e**–**g** IgG_1_ and IgG_2c_ isotype titers were determined by reciprocal endpoint titers against each antigen using anti-mouse IgG_1_ or IgG_2c_ antibodies, respectively. All antibody data are expressed as mean ± SEM of results from at least 5 mice per group and analyzed in triplicate. Significant differences between IgG_1_ and IgG_2c_ titers were determined via Student’s *t* test. *p < 0.05, ****p < 0.0001.

To evaluate the immunogenicity of the different AuNP-coupled glycoconjugates and assess the protection differences afforded by different antigens, we evaluated the antigen-specific humoral responses associated with the highest protection against *Bm* lethality. Animals immunized with each individual formulation containing OpcP, OmpW, or HA had total IgG of ≥10^7^ endpoint titers against each antigen (Fig. 4a–c). In addition, animals that received the AuNP-Combo2-LPS formulation, containing equivalent amounts of OpcP, OmpW, and HA, showing robust responses against each antigen, which were equivalent to single protein-LPS formulations (Fig. 4a–c). Further, animals immunized with either AuNP-OmpW-LPS, AuNP-HA-LPS, AuNP-OpcP-LPS, as well as with AuNP-Combo2-LPS, maintained robust humoral responses against LPS with the highest titers of 10^6^, and AuNP-OmpW-LPS associated with the lowest 10^5^ endpoint titers (Fig. 4d). To evaluate the differences in immune polarization or functionality, we measured the isotype changes in single-protein formulation after vaccination (Fig. 4e–g). We observed robust Th1-biased immune response with statistically higher levels of IgG_2c_ in animals immunized with OpcP- and OmpW-formulations (Fig. 4e, f). These results indicate the ability of intranasally immunization with AuNP-delivered glycoconjugates to induce robust antigen-specific antibody titers and a Th1-biased immune response that correlated with protection.

### Serum from AuNP-glycoconjugate vaccination is associated with a reduction in bacterial adherence and increased opsonophagocytosis linked to bacterial death

**Fig. 5:**
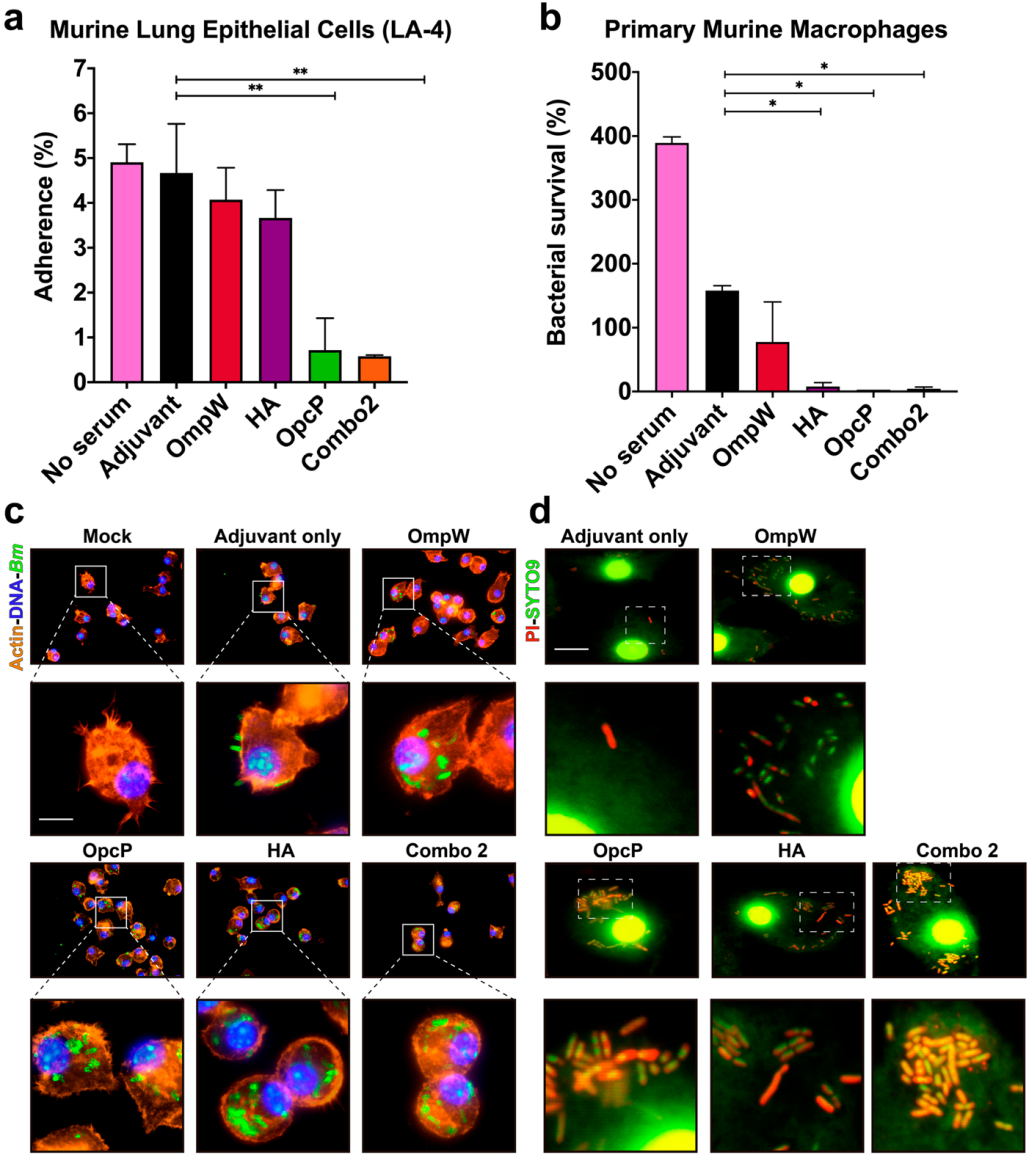
Sera from surviving animals reduce adherence to mouse lung epithelial cells and promote bacterial opsonophagocytosis by primary murine macrophages, leading to bacterial death. *Bm* 23344 bacterial cells (5 × 10^6^ CFU) were incubated in the presence or absence of immunized serum (10% of final volume) from animals immunized with each vaccine group (pooled from at least 5 animals) for 1 h at 37 °C. Serum from naive mice (adjuvant-only) served as controls. After incubation, bacteria were used to infect **a** LA-4 cells or **b** primary murine macrophages for  1h and  2h, respectively. After infection, LA-4 cell monolayers or primary macrophages were processed to enumerate adhered or surviving bacteria, respectively. All data are expressed as mean ± SEM of results from two independent experiments using sera from *n* = 5 mice per group. Significant differences were determined via one-way ANOVA followed by Tukey’s post hoc test compared to the Adjuvant-only group (*p < 0.05, **p < 0.001). **c** Fluorescence microscopy analysis of primary murine macrophages after *Bm* 23344 infection in the presence of immune serum (from vaccinated groups). After infection, cells were fixed, permeabilized, and stained with phalloidin-rhodamine (actin), DAPI (bacteria and cell nuclei), and examined by immunofluorescence (sera anti-LPS followed by a rabbit anti-mouse Alexa Fluor-488). **d** LIVE/DEAD^TM^ *Bac*Light^TM^-stained primary murine macrophages infected with *Bm* 23344 in the presence of sera from each immunization group as described above. Panels below each group represent magnifications (10×) of the images on top. Images were taken using an Olympus BX51 upright fluorescence microscope (60×) and processed using Image J software. Scale bars 25 μm.

The differences in functionality between the different vaccine groups were analyzed by evaluating the ability of serum from immunized mice to block bacterial adherence onto epithelial cells or to increase macrophage-mediated phagocytosis. Bacteria in the presence of sera from AuNP-OpcP-LPS or AuNP-Combo2-LPS had a significant reduction in adherence to murine lung epithelial cells, compared to bacterial infection without sera (Fig. 5a). The other sera did not significantly interfere with the bacterial adherence properties. In contrast, by 2 h post infection, bacteria in the presence of sera from AuNP-OpcP-LPS, AuNP-HA-LPS, and AuNP-Combo2-LPS immunized groups were associated with significantly lower levels of surviving *Bm* in primary mouse macrophages in comparison to bacteria in the presence of naïve sera (adjuvant-only group) (Fig. 5b). In the presence of serum from AuNP-OpcP-LPS, AuNP-HA-LPS, and AuNP-Combo2-LPS, we observed an increase in the number of internalized bacteria by primary murine macrophages by 1 h post infection, comparing to bacteria in the presence of serum from naïve animals (Fig. 5c). In addition, using a live/dead bacterial stain, we were able to visualize the viability of intracellular bacteria in primary macrophages by assessing the loss of membrane integrity via the incorporation of propidium iodide (Fig. 5d). In the presence of sera from the immunization groups AuNP-OpcP-LPS, AuNP-HA-LPS, and AuNP-Combo2-LPS, we noticed a greater proportion of internalized bacteria by macrophages had compromised membranes, as visualized in red within the bacterial cytoplasm (Fig. 5d). These results confirm that the antibodies in the serum from AuNP-protein-LPS-immunized animals block bacterial adherence to lung epithelial cells and enhance antibody-mediated phagocytosis by macrophages. More importantly, we showed that both effects are present in serum from animals immunized with AuNP-Combo2-LPS formulation.

## Discussion

Efficient delivery of antigens by AuNPs requires a stable association with the AuNPs interface^20^. This nanovaccine platform has been used for the delivery of a variety of biomolecules and in vaccine delivery, and it has been shown to help deliver protein antigens and enhance protection. Although the exact mechanism by which this may occur is not entirely understood, it is believe that nanoparticles may act as adjuvants to enhance antigen stability, improve the ability to cross mucosal barriers, and for antigen presentation upon antigen-presenting cell internalization^12,13,21^. A recent study showed that upon antigen delivery and dendritic cell internalization, OVA-conjugated AuNPs showed enhanced OVA-specific polyfunctional T cells responses, including the induction of CD8^+^ cytotoxic T cells^15^. In addition, the mechanism of nanoparticle uptake to draining lymphatic organs is more efficient compared to soluble antigens-alone, an effect dependent on the NP’s physicochemical characteristics, such as their size, shape, or charge^14,15,21,22^. Based on these premises, we covalently linked each recombinant predicted antigen to the surface of AuNPs by a covalent modification via a small hydrophobic linker (16-MHDA). To exploit the stability, loading efficiency of AuNPs, and the multivalent display of antigens, we conjugated the LPS of *Bth* containing both the O-antigen molecule (OAg) as well as the lipid A moiety onto the protein-decorated NPs, to expand the protective response against *Bm*. The LPS of *Bth* has been demonstrated to have immune and adjuvant stimulatory activity^23^. To induce antibody responses against the LPS moiety of *Bth*, a well-known T cell-independent antigen, we covalently coupled LPS to proteins on the surface of AuNPs to engage T cell help and allowing for memory B cell development, and long-lived T cell memory^24^. In addition, the final vaccine formulation contained 20 μg of CpG ODN 2395, a toll-like receptor 9 (TLR9) agonist shown to help induce a Th1-biased response and a therapeutic molecule in helping control *Bm*-infection during early times post infection^25,26^. Together, the function of AuNPs as antigen delivery vehicles along with a Th1-biased adjuvant could help augment and divert the immunogenicity of the protein and LPS antigens. As predicted, we were able to show the synthesis, by covalent coupling of both the protein and LPS antigens, to the surface of a single AuNP by using UV-Vis and TEM.

Given that inhalation of *Bm* is a potential exposure route, a vaccine that elicits robust mucosal responses is expected to provide the most efficient protection against aerosol infection. Most vaccines platforms that are delivered by the parenteral route fail to induce strong mucosal responses^13^. Therefore, intranasal vaccination is an efficient and appealing strategy for the induction of mucosal-specific immunity^13^. Among the multiple challenges hindering the development of nasal vaccination are the inefficient antigen uptake, rapid antigen clearance, size-restriction across epithelial barriers, and safe use of adjuvant for intranasal delivery^13^. Nanoparticle-based vaccines offer a mucosal-adjuvant strategy that is safe and efficient for antigen delivery, uptake, and processing^12,15,16^. Using a murine model of glanders, we evaluated six bio- and immuno-informatically predicted antigens for their protective ability against a low-dose challenge. We found that three new antigens provide significant protection against such inhalational challenge. OpcP and OmpW are two predicted porins that are among the most abundant proteins in the outer membrane of *Bpm* and *Bm*^27^. Hemagglutinin is a predicted member of the autotransporter family of proteins with an unknown active function in the pathogenesis or life cycle of *Bm*. Our results indicated that these antigens alone provided significant protection against a low-dose *Bm* intranasal challenge. In addition, these candidates provided a high degree of protection against a high-dose challenge of *Bm*, with the highest protection afforded by OpcP and OmpW, two proteins present in a wide range of growth conditions in both *Bpm* and *Bm*^27^. In addition, a combination vaccine formulation of the three most-protective antigens (AuNP-Combo2-LPS) provided 100% survival against a high-dose *Bm* challenge, although no observable difference was seen when delivered in combination with three other proteins (FlgL, Hcp1, and OpcP1). Plausible justifications for this observation might be that the protective antigens in a formulation containing all six candidates are diluted, not reaching the appropriate concentration, in comparison to the formulation containing only three antigens. Another option is that the protective responses to those antigens are out-competed in the combination formulation containing all six antigens (AuNP-Combo1-LPS). Future studies should focus on determining the optimal antigen dose and combination that provide the most effective protection against *Bm* colonization in the lungs, liver, and spleen, while maintaining establishment of an acute infection.

Our results demonstrated that AuNP-conjugated glycoconjugates induce strong antigen-specific humoral responses when delivered intranasally. Interestingly, in vaccine groups with the highest survival rate, the total IgG titers remained equivalent when the antigen was delivered alone or in combination with another two antigens. In addition, we also observed elevated LPS-specific humoral responses, with the highest levels seen in the AuNP-Combo2-LPS formulation. We have shown that AuNP-protein-LPS delivery is able to maintain elevated LPS-specific titers without affecting the induction of strong protein-specific responses. Studies have shown high degree of structural similarity of the LPS in *Bth*, *Bpm*, and *Bm* as well as their function as potent innate immune stimulator^23,28^. Although the role of LPS-specific humoral responses in human glanders patients is unknown, in the case of melioidosis patients, the O-polysaccharide (OPS)-specific titers correlate to higher degree of survival in convalescent patients^29^. Importantly, convalescent human melioidosis patients appear to have higher LPS-specific IgA titers, compared with non-survivors^29^. Our results further strengthen this hypothesis by demonstrating the importance of serum IgG against both protein and LPS antigens at providing protection against inhalational *Bm* infection. In addition, we previously showed a similar observation with two known protective *Bm* antigens, Hcp1 and FliC, when delivered in a AuNP-glycoconjugate model showing the best protection afforded upon conjugation of the LPS structure to a protein carrier^30^.

Given the complex role of serum antibodies in mediating antibacterial responses, we also analyze the differences in antibody function in the different vaccine groups. While the C57BL/6 mouse genetic background does not express the locus for the IgG_2a_ antibody subtype, rather IgG_2c_, we measured this antibody isotype to prevent an incorrect interpretation of murine humoral immune responses^31,32^. Our results suggest that the higher IgG_2c_ levels correspond to a Th1-biased immune response, potentially implicating cellular-mediated immune responses as mediators of vaccine protection. However, future studies should focus in the different AuNP-glycoconjugates an assess how cellular-mediated immunity is elicited. To further analyze the potential effector function associated with the humoral responses, we decided to measure in vitro two other important antibody functions. We evaluated the bacterial adherence to murine lung epithelial cells in the presence or absence of serum from each vaccine group and found a significant decrease in bacterial adherence with sera from the OpcP-LPS and Combo-LPS vaccine groups. The data suggest that serum inhibition of bacteria adherence is due to the antibodies against OpcP, an such inhibitory effect is retained in the combination vaccine formulation. This bacterial protein is highly expressed on the surface of *Bm* and across a wide range of conditions and represents an efficient mechanism of blocking adherence to prevent internalization and to prevent full *Bm* pathogenesis. We also used primary mouse macrophages to define whether serum enhanced bacterial uptake and found that this phenotype was maintained in most vaccine groups, including the combination vaccine formulation (Combo2). The decrease in bacterial viability, as seen by reduced bacterial survival, and visualization of compromised bacterial membranes within macrophages treated with the AuNP-OpcP-LPS, AuNP-HA-LPS, and AuNP-Combo2-LPS sera indicates that serum acted during bacterial uptake but also during bacterial killing. Lastly, these results indicate that antibodies against different proteins have a variety of anti-*Bm* properties and the combination vaccine formulation retained these different effector functions. Our results offer a possible explanation for the observed enhanced protection in vivo against inhalational glanders. Furthermore, these results indicate that a multivalent vaccine against *Bm* may provide enhance and complete protection against infection and colonization. Overall, our study highlights the identification of three new protective subunit vaccine candidates against *Bm* which warrant further investigation into the most effective formulation dose and/or combination that can be advance to human trails.

## Materials and methods

### Bacterial strains and growth conditions

*Burkholderia thailandensis* (*Bth*) E264 and *B. mallei* (*Bm*) ATCC 23344 (China 7) were routinely grown aerobically at 37 °C in Luria-Bertani (LB) medium containing 1% NaCl or LB medium supplemented with 4% glycerol (LBG), respectively. All chemical reagents, unless otherwise noted, were purchased from Sigma-Aldrich.

### Cloning and protein purification

Open reading frames (ORFs) from each candidate amplified from genomic DNA from *Bm* 23344: *hcp1* (BMAA0742), *ompW* (BMA2010), *opcP* (BMAA1353), *opcP1* (BMAA1122), *flgL* (BMA3336), and Hemagglutinin-family protein (HA; BMAA1324) were cloned in pET30a(+) expression vector with a 6× histidine (His) tag positioned in the C-terminus^18^. Briefly, in-frame genes of each candidate were amplified from purified *Bm* 23344 genomic DNA using the restriction enzymes NdeI and XhoI and the plasmids were transformed into competent *Escherichia coli* BL21 (DE3) (New England BioLabs). To induce protein expression, overnight cultures were diluted 1:20 in 1 L of Luria Bertani (LB) broth containing 50 μg/mL of kanamycin, grown to an optical density at 600 nm (OD600) between 0.6 and 0.8, and induced with 1 mM (final concentration) of isopropyl-D-1-thiogalactopyranoside (IPTG). Cultures were centrifuged (4000*g* for 15 min) after 3 h post-induction, and each resulting bacterial pellet was frozen at –20 °C. The bacterial pellets were then resuspended in 40 mL of 1× Dulbecco’s phosphate- buffered saline (DPBS) containing 10% glycerol and 25 mM sucrose with a 1 mg/mL final concentration of lysozyme and 0.2% sodium deoxycholate and a tablet of cOmplete EDTA-free protease inhibitor cocktail (Roche, Germany). This lysate was then sonicated, centrifuged, and the pellet was used for subsequent washes to maximize soluble protein extraction. After spinning down at 20,000 × *g* for 40 min, the supernatant was subjected to sterilization using a 0.2-μm pore size filter (Millipore). Soluble protein extracts were then bound to Talon® cobalt (Co^2+^) columns (GE Healthcare, USA) and washed with PBS buffer–50 mM imidazole. Proteins were eluted from affinity columns by applying a 1× PBS buffer with 10% glycerol, 25 mM sucrose and 250 mM imidazole. Fractions containing soluble protein were collected and pooled before dialysis into PBS containing 10% glycerol and 25 mM sucrose overnight at 4 °C. Endotoxin levels were tested using a Pierce LAL chromogenic endotoxin quantification kit (ThermoFisher Scientific, USA) following manufacturer’s specifications. The limit of detection for endotoxin is approximately 0.1 EU/mL of solution. The purified proteins and protein standards were subjected to a colorimetric bicinchoninic acid assay (BCA) in accordance with the manufacturer’s protocol and were then stored at −80 °C until use (Pierce^TM^ Protein Assay Kit, ThermoFisher Scientific). For protein visualization, 0.25 μg of each protein was run on SDS-PAGE gel by electrophoresis. Gels were transferred to a nitrocellulose membrane for Western blot analysis. A mouse anti-histidine antibody (Invitrogen™ Catalog No. R930-25) was used (1:5,000) and the reaction mixture incubated overnight at 4 °C, followed by horseradish peroxidase (HRP)-conjugated rabbit anti-mouse IgG (Southern Biotech™ Catalog No. OB1030-05) was used (1:10,000) as a secondary antibody. Protein bands were visualized by adding ECL substrate (ThermoFisher Scientific), and the results were imaged on film. Molecular weight markers were visualized using Precision Plus Protein^TM^ WesternC^TM^ Blotting Standards (Bio-Rad) with StrepTactin-HRP conjugate (Supplemental Fig. 1). WB and Coomassie-stained gel were generated from the same experiment and processed in parallel.

### Lipopolysaccharide extraction

The LPS from *Bth* E264 was isolated by the hot phenol-extraction method^18,28^. Briefly, a pellet of 4 L of LB-grown *Bth* to stationary phase (24 h at 37 °C and 200 RPM) was collected (6000*g* for 15 min) and lysed in the presence of a mixture of 1:1 phenol in water (ThermoFisher Scientific). After lysis at 80 °C, phenol was removed by dialysis 4× into ultra-pure water and centrifuged (15 min at 6000*g*) to clarify the solution, and the supernatant was lyophilized. The lyophilized solution was resuspended in an aqueous solution containing 10 mM Tris-HCl (pH 7.5), 1 mM MgCl_2_, 1 mM CaCl_2_ and digested with RNase, DNase I and Proteinase K (50 μg/mL each). After clarification (100,000*g* for 3 h), the resulting supernatant containing LPS was lyophilized. The sample containing the LPS was washed 5× times with 90% ethanol and lyophilized. After lyophilization, the pellet was weighted, resuspended in PBS, and stored at −80 °C until use. The purity of LPS was assessed by SDS-PAGE electrophoresis, followed by Silver Staining following manufacturers protocol (Pierce^TM^ Color Silver Stain Kit).

### Gold nanoparticle synthesis and coupling

Spherical 15 nm gold nanoparticles (AuNPs) were synthesized by the Turkevich method^19^. Briefly, 1 mM gold (III) chloride trihydrate underwent a reduction reaction with 90 mM sodium citrate dihydrate. Particle size and shape was analyzed by transmission electron microscopy (TEM). To stabilize the conjugation of soluble antigens onto the AuNP surface, 0.1 mM 16-mercaptohexadecanoic acid (16-MHDA) and 0.1% Triton X-100 were added to AuNPs. After 2 h of incubation, this solution was filtered by centrifugation (EMB Millipore Amicon^TM^ Ultra-15, 30 kDa molecular weight cutoff [MWCO]), and the procedure was repeated for 2 h to ensure complete coverage. Covalent protein conjugation by carbodiimide synthesis^33^ was achieved by the addition of 20 μg of protein per mL for maximum coating of nanoparticles (previously defined in^33^, by SDS-PAGE densitometry analysis) in the presence of 1 mM DMTMM [4-(4,6-dimethoxy-1,3,5-triazin-2-yl)-4-methyl-morpholinium chloride]. The AuNP-protein conjugation reactions were carried out in 100 mM borate buffer for 12 h. Attachment of 16-MHDA and protein was confirmed by measuring plasmon resonance via UV-Vis spectroscopy, TEM, as well as by SDS-PAGE. To conjugate LPS onto the AuNP-protein conjugates, we employed the thiol-maleimide synthesis mechanism. To achieve this, LPS was activated by the addition of 80 mM EMCH (N-(ε-maleimidocaproic acid hydrazide) cross-linker in the presence of 40 mM EDC (*N*-(3-Dimethylaminopropyl)-*N*′-ethylcarbodiimide hydrochloride) and 10 mM NHS (*N*-hydroxysuccinimide) in 50 mM MES buffer (pH 7.0). After 1 h at room temperature, LPS was concentrated to desired concentration using Amicon^TM^ Ultra-15, 30 kDa MWCO. After desalting the LPS in 5 mM EDTA, 20 μg of activated LPS were added per mL of protein-coupled AuNPs previously activated in the presence of 250 μM SATA (S-acetylthioglycolic acid *N*-hydroxysuccinimide ester) for 1 h at room temperature. After 4 h of incubation, the reaction was quenched with 5 mM N-ethylmaleimide. The AuNP-protein-LPS conjugates were washed 2× with PBS containing 10% glycerol and 25 mM sucrose and concentrated to desired volume containing a concentration of ∼ 0.23 μg/μL of both protein and LPS using an Amicon® Stirred Cell containing a 100 kDa MWCO filter.

### Ethics statement

All manipulations of *Bm* were conducted in CDC/USDA-approved and registered biosafety level 3 (BSL3) facilities at UTMB in accordance with approved BSL3 standard operating practices. The animal studies were carried out humanely in ABSL3 facilities in strict accordance with the recommendations in the Guide for the Care and Use of Laboratory Animals by the National Institutes of Health. The protocol (IACUC #0503014D) was approved by the Institutional Animal Care and Use Committee at UTMB.

### Animal studies

Female 6-to-8-week-old C57BL/6 mice were purchased from Jackson Laboratories (Bar Harbor, ME, USA). Animals were housed in microisolator cages under pathogen-free conditions with food and water available *ad libitum* and maintained on a 12-h light cycle. To allow adequate acclimation, mice were housed within the animal facility for 1 week prior to experimentation.

### Immunization and challenge studies

C57BL/6 mice (*n* = 9 per group) were inoculated intranasally (i.n.) three times in 2-week intervals with 50 μL formulations. Animals received each of the AuNP-protein-LPS conjugate. Two combination AuNP formulations were synthesized: AuNP-Combo1-LPS (containing Hcp1, OmpW, OpcP, OpcP1, FlgL, and HA) or AuNP-Combo2-LPS (containing HA, OmpW, and OpcP). Each vaccine formulation contained a total of approximately 10 μg of protein, 10 μg LPS, along with 20 μg of CpG ODN 2395 (InvivoGen, USA). Control groups received 20 μg of adjuvant alone. To evaluate antibody titers, blood was drawn retro-orbitally 2-weeks following the last boost (*n* = 5). To isolate sera, blood was incubated for 30 min at room temperature (RT) to allow clotting and centrifuged (10,000*g* for 10 min). Sera was removed and stored at –80 °C until use. For assays requiring serum, the samples from immunized animals (*n* = 5) were pooled and stored. Three-weeks after administering the last immunization, animals were i.n. challenged with a low- or high-dose challenge of *Bm* 23344 in 50 μL formulations. The low dose challenge animals received 2 LD_50_ (2.8 × 10^4^ CFU per mouse), while the high-dose challenge received 50 LD_50_ (7 × 10^5^ CFU per mouse). To enumerate bacterial colonization, the lung/spleen (low-dose challenge) or lung, liver, and spleen (high-dose challenge) of mice were collected. Organs were homogenized in 1 mL of 1× PBS, serially diluted, and plated on LBG agar and incubated at 37 °C for 48 h. The bacterial limit of detection (LOD) was determined to be 1 CFU/organ.

### Detection of antigen-specific antibodies

Baseline and post-vaccinated sera were collected from animals administered with adjuvant only, individual AuNP-protein-LPS conjugates, and from AuNP-Combo-LPS formulation two-weeks after the second boost. Whole blood was collected via retro-orbital bleeding and stored in Microvette tubes without anticoagulant. The sera were separated by centrifugation and stored at –80 °C. The protein-specific total IgG, IgG_1_, and IgG_2c_ titers were determined by indirect enzyme-linked immunosorbent assay (ELISA)^17^. Briefly, a microplate (Costar, Cambridge, MA) was coated with each protein or LPS antigen (1 μg/well) in a mixture with 1× PBS (Corning, USA) and maintained overnight at 4 °C. Wells were washed twice with washing buffer (0.05% Tween 20—DPBS) and then blocking buffer (0.05% Tween 20, 2% bovine serum albumin [BSA], 1×DPBS) was added at RT for 2 h. The blocked wells were washed twice before the addition of sample diluent (1% BSA—0.05% Tween 20—1× DPBS). The sera were added to each top dilution well in triplicate, and 2-fold dilutions were performed following incubation at RT for 2 h. Diluted goat anti-mouse IgG-HRP (Southern Biotech™ Catalog No. OB1030-05), IgG_1_-HRP (Southern Biotech™ Catalog No. 1070-05) or IgG_2c_-HRP (Southern Biotech™ Catalog No. 1077-05) (1:5,000) was added into each well and then incubated for 3 h after washing. Plates were washed four times prior to addition of tetramethylbenzidine substrate solution (Invitrogen, USA). Stop solution (2 N H_2_SO_4_) was added, and the samples were immediately read at 450 and 570 nm using a microplate reader (BioTek, USA). The results were reported as the reciprocal of the highest titer, giving an optical density (OD) reading of at least the mean ≥2 standard deviations compared to the baseline sera. All assays were performed in triplicate, and results were reported as mean reciprocal endpoint titers.

### Serum adherence inhibition assays

Murine respiratory alveolar epithelial cells LA-4 (American Type Culture Collection (ATCC^®^) CCL-196^TM^) were maintained at 37 °C with 5% CO_2_ in complete F-12-Kaigh’s (F12-K) medium (Gibco, USA). Complete F12-K minimum was supplemented with penicillin and streptomycin (100 U/mL and 100 μg/mL, respectively), and 10% fetal bovine serum. For adhesion assays, 12-well plates were seeded with 5×10^5^ cells/per well. At approximately 1 h prior to infection, the monolayer was washed twice with 1 mL PBS prior to addition of 1 mL of medium containing no supplements. Bacterial inoculum (input) were prepared from 16 h cultures of *Bm* 23344 and adjusted to an MOI of 10 (5 × 10^6^ CFU) and incubated in the presence or absence of immune serum from individual AuNP-protein-LPS, AuNP-Combo2-LPS, adjuvant-only serum, or naive sera (final concentration of 10%) for 1 h at 37 °C with slight agitation. After incubation in the presence of sera, bacteria were collected in 1 mL of fresh media and used to infect cell culture plates containing 5 × 10^5^ cells. Monolayers were incubated for 1 h at 37 °C with 5% CO_2_. After incubation, cells were washed three times with PBS prior to the addition of 100 μL of 0.1% Triton X-100 in PBS. After detachment, the cells were serially diluted in PBS and plated on LBG agar plates for 48 h to enumerate the number of adhered bacteria (output). The percentage of adhered bacteria was determined as the output/input × 100. Data represent results of two independent experiments performed using pooled sera from (*n* = 5) mice.

### Macrophage survival assay and fluorescence microscopy

C57BL/6 murine bone marrow-derived primary macrophages (BMDM) (Cat No. C57-6030, Cell Biologics Inc., Chicago) were routinely grown in complete primary cell culture medium following manufacturer’s instructions (Cat No. M3368, Cell Biologics, Chicago). Cells were incubated at 37 °C and 5% CO_2_. For infection and microscopic analysis, 5 × 10^5^ cells were grown in 12-well cell-culture grade plates (Corning) or round cover slips and incubated overnight, prior to treatment. Bacterial inoculum used at an MOI of 10 (5 × 10^6^ CFU) was incubated in the presence or absence of immune serum from AuNP-protein-LPS, AuNP-Combo2-LPS, or naive sera (final concentration of 10%) for 1 h at 37 °C with slight agitation. After incubation in the presence or absence of sera, bacteria were collected in 1 mL of fresh media and used to infect cell culture plates containing 5 × 10^5^ cells. For cell infection, monolayers were incubated for an additional 2 h at 37 °C with 5% CO_2_. After infection, cells were either washed, lysed with 0.1% Triton X-100 in PBS, and plated in LBG agar for 48 h, or fixed with 4% paraformaldehyde in PBS for 30 min. Cells were permeabilized with 0.1% Triton X-100 for 5 min and polymerized actin and DNA were visualized using rhodamine isothiocyanate-phalloidin (Molecular Probes-Invitrogen, USA) or DAPI (Molecular Probes-Invitrogen, USA), respectively. *Bm* 23344 cells were detected with serum from *Bth* LPS-immunized mice (1:500), followed by a goat anti-mouse IgG, IgM (H + L) secondary antibody conjugated to Alexa 488 (ThermoFisher Scientific™ Catalog No. A-10667) (1:10,000). Cells were mounted using ProLong Gold Antifade (Molecular Probes-Invitrogen, USA) prior to visualization. Images were examined using an Olympus BX51 upright fluorescence microscope and analyzed using ImageJ software, National Institutes of Health^34^.

### Visualization of live and dead intracellular bacteria

Primary murine macrophages (5 × 10^5^) were infected as described above with a MOI of 10, for  2 h, in the presence of serum from immunized animals. Prior to infection, bacteria were incubated in the presence of sera for 1 h and input bacteria was quantified. After infection, cells were slightly permeabilized with 0.1% saponin in PBS for 10 min at room temperature. Cells were then stained with LIVE/DEAD^TM^ *Bac*Light^TM^ Kit (Molecular Probes, Life Technologies) containing propidium iodide (PI) or SYTO 9, following the manufacturer’s instructions. Following staining, cells were washed three times with PBS and fixed with 4% PFA for 20 min. Cells were directly mounted using ProLong gold antifade (Molecular Probes, Life Technologies) and visualized using an Olympus BX51 upright fluorescence microscope and analyzed using ImageJ software, National Institutes of Health^34^.

### Statistical analysis

All statistical analysis was done using GraphPad Prism software (v 8.0). *p-*Values of <0.05 were considered statistically significant. Quantitative data are expressed as means ± standard errors. All data were analyzed for normality before the corresponding test was run. Results of colonization, antibody, serum adherence inhibition, and opsonophagocytosis assays were analyzed by one-way analysis of variance (ANOVA) using Tukey’s post hoc test or the Kruskal–Wallis post hoc test when data were not normally distributed. Significant differences between IgG_1_ and IgG_2c_ titers were determined via Student’s *t-*test. Statistical differences in survival were determined by the Kaplan–Meier method, followed by log-rank test. Levels of significance compared to the adjuvant-only group: *p < 0.05, **p < 0.005, ***p < 0.0005, ****p < 0.0001.

### Reporting summary

Further information on experimental design is available in the Nature Research Reporting Summary linked to this paper.

## Supplementary information

Supplemental Material

Reporting Summary Checklist FLAT

## Data Availability

The authors declare that the data and unique biological materials supporting the findings of this study are available within the paper (and its supplementary information files) or will be readily provided by the authors.
