# Supplementary material for: Multicomponent gold nano-glycoconjugate as a highly immunogenic and protective platform against Burkholderia mallei
Source: NPJ Vaccines. 2020 Sep 10;5:82. doi: 10.1038/s41541-020-00229-9 (PMC7483444; doi:10.1038/s41541-020-00229-9)
Supplement: Supplementary file 1 — Supplemental Material [file 41541_2020_229_MOESM1_ESM.pdf]

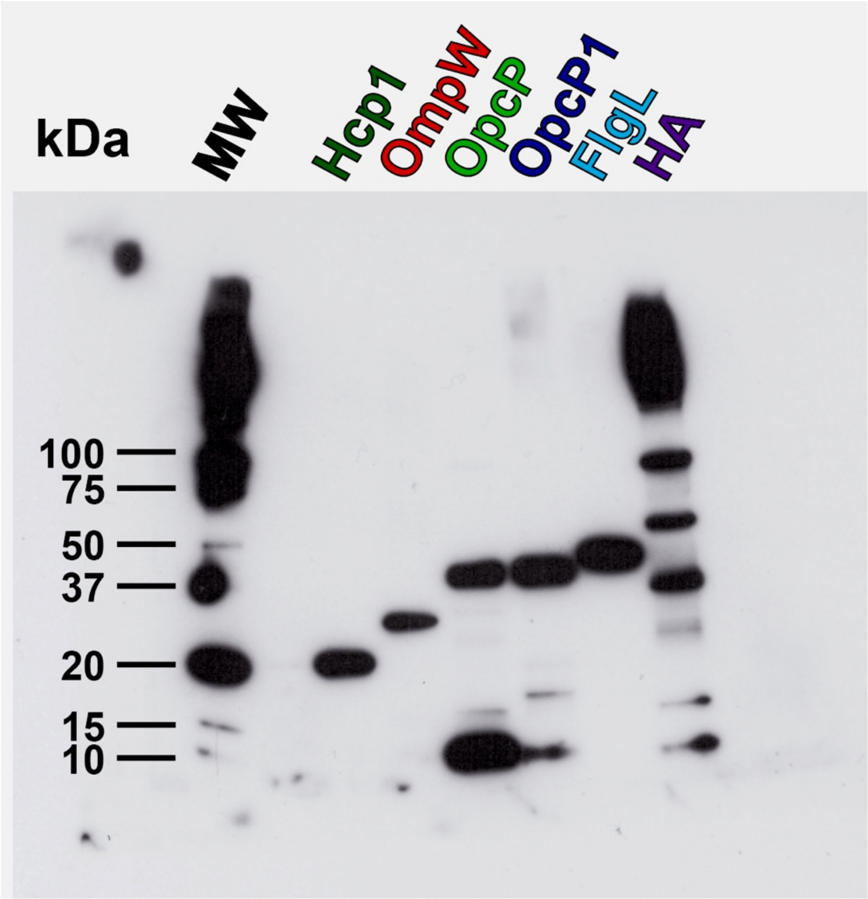

Supplementary Figure 1. Complete western blot of vaccination candidates. Western blot of recombinantly expressed protein antigens after purification, using 0.25 μg of each individual candidate: Lane 1; Molecular weight marker, lane 2; Hcp1 (18 kDa), lane 3; OmpW (29 kDa), lane 3; OpcP (39 kDa), lane 4; OpcP1 (41.6 kDa), lane 5; FlgL (42 kDa), and lane 6; Hemagglutinin (HA) (79.6 kDa). Molecular weight marker was visualized using Precision Plus Protein™ WesternC™ Blotting Standards with StrepTactin-HRP conjugate.
